# Comparison of FIB-4 and transient elastography in evaluating liver fibrosis of chronic hepatitis C subjects in community

**DOI:** 10.1371/journal.pone.0206947

**Published:** 2018-11-07

**Authors:** Pin-Nan Cheng, Hung-Chih Chiu, Yen-Cheng Chiu, Shu-Chuan Chen, Yi Chen

**Affiliations:** 1 Division of Gastroenterology and Hepatology, Department of Internal Medicine, National Cheng Kung University Hospital, College of Medicine, National Cheng Kung University, Tainan, Taiwan; 2 Public Health Bureau, Tainan City Government, Tainan, Taiwan; National Taiwan University Hospital, TAIWAN

## Abstract

**Background and aim:**

The role of non-invasive methods to evaluate fibrosis severity of chronic hepatitis C (CHC) subjects in community needs to be explored. This study investigated FIB-4 and transient elastography (TE) in staging liver fibrosis of CHC subjects in community.

**Methods:**

A total of 905 subjects who were positive for anti-HCV antibody from five districts of Tainan City of Taiwan were invited to participate in surveillance activities for CHC. FIB-4 and TE were measured for each participant.

**Results:**

A total of 502 subjects with detectable HCV RNA and valid TE were enrolled. The distribution of FIB-4 and TE values differed markedly. Both methods exhibited a strongest correlation in subjects with at age 50~60 years (r = 0.655, p <0.001). FIB-4 score increased proportionally with age (p <0.001), but TE did not (p = 0.142). The intraclass correlation efficient of both methods was 0.255 (p <0.001). Subjects with TE defined advanced fibrosis exhibited younger age, higher BMI, higher platelet count, lower FIB-4 score, higher incidence of fatty liver and splenomegaly, and higher controlled attenuation parameter value than those defined by FIB-4. By multivariate logistic regression analysis, higher ALT levels, higher incidence of fatty liver, and presence of splenomegaly were the independent factors associated with advanced fibrosis defined by TE rather than defined by FIB-4.

**Conclusions:**

FIB-4 and TE defined different distribution of fibrosis stages in same HCV population. FIB-4 was deeply influenced by age whereas TE was not. TE had the advantages over than FIB-4 in strong association with splenomegaly and in detecting the role of non-alcoholic fatty liver disease in advanced fibrosis.

## Introduction

More than 170 million people are infected with hepatitis C virus (HCV). Chronic HCV infection leads to the development of chronic hepatitis, liver cirrhosis and associated complications, hepatocellular carcinoma, and liver-related mortality [1–3]. Treatment to eradicate HCV provides benefits with respect to both liver- related or extrahepatic morbidity and mortality [4]. In era of pegylated interferon/ribavirin, barriers to treatment include fear of the side effects of drugs, ineligibility for treatment, intolerance of treatment, and others [5]. Direct acting antiviral (DAA) therapy is a more efficacious, shorter-duration, and well-tolerated treatment without most of the barriers to treatment by pegylated interferon/ribavirin therapy [6,7].

Patients with CHC and advanced fibrosis have a higher incidence of cirrhosis-related complications and are more likely to need a liver transplant [8–10]. In cases of advanced fibrosis, the benefits of antiviral treatment are observed after HCV has been eradicated [11,12]. Hence, identifying CHC patients with advanced fibrosis is clinically important for prioritizing treatment. Ideally, all CHC patients should be treated. However, the huge economic burden on care providers or the government makes this ideal goal difficult to achieve. A strategy for treating CHC patients sequentially in a manner that accounts for the severity of liver fibrosis is reasonable, evidence-based, and suggested in the EASL HCV treatment guidelines [13]. The two widely used non-invasive methods for identifying and classifying a stage of fibrosis are FIB-4 and the measurement of liver stiffness by transient elastography (TE). FIB-4 has been demonstrated to have high negative and positive predictive rates of advanced fibrosis [14]. The measurement of liver stiffness by transient elastography is quite different from FIB-4 and depends on a vibration-generating machine to apply vibrations to the liver and then obtain the propagation velocity of shear wave [15]. Based on the fundamental difference between the FIB-4 and TE methods, analyzing the features of a CHC population with various stages of fibrosis is of interest.

In Tainan City, hepatitis B virus and HCV infections are endemic [16,17]. The application of non-invasive methods, such as FIB-4 and TE, to evaluate the severity of fibrosis in HCV subjects is an important first step in referral for DAA treatment. Understanding the advantages and limitations of FIB-4 and TE is required for further and future applications in community.

This community-based study used FIB-4 and TE to evaluate the severity of liver fibrosis, especially advanced fibrosis, and to analyze the characteristics of CHC subjects.

## Materials and methods

### Surveillance activities of CHC

Since 2017, the National Institute of Health of the government of Taiwan has been reimbursing DAA therapy for CHC. Only patients with advanced fibrosis stage 3 or stage 4 which was defined by histological evaluation, by TE (with liver stiffness ≥9.5kPa; Echosense, France), or by FIB-4 score >3.25 were qualified for DAA treatment. To identify subjects that satisfy the conditions for the reimbursement of DAA therapy for CHC in community, surveillance activities were conducted in cooperation with the Public Health Bureau of the Tainan City Government in the five districts of Tainan City (Rein-De, Jiang-Chun, Qui-Ren, Hsin-Hwa, and Liuo-Jia). Before surveillance activity, candidate of subjects were informed in detail about the surveillance activities, including their dates, locations, and items of examination, and required information by officials of the health departments of local districts and the Tainan City Government. The items of surveillance activity included baseline characteristics, measurement of body weight/body height/waist circumference, blood tests, abdominal sonography, and measurement of liver stiffness by TE. The Research Ethics Committees of National Cheng Kung University Hospital approved this study, which was carried out according to the guidelines of the International Conference on Harmonization for Good Clinical Practice. All patients provided written informed consent before participation.

### Patients

In the past ten years, the Tainan City government has performed screening activities for viral hepatitis in all districts of Tainan City. Subjects participated in a screening program from January 2015 to January 2018 in the five districts and those who were positive for anti-HCV antibody were the target population.

### Examinations

Blood samples were collected and stored at -70°C until testing. The blood tests included aspartate aminotransferase (AST), alanine aminotransferase (ALT), complete blood counts, quantitation of HCV RNA, and genotyping of HCV in HCV RNA-positive cases. Quantitation and genotyping of HCV RNA were performed using the real-time polymerase chain reaction (Abbott RealTime HCV quantitative assay; Abbott Molecular Inc., IL, USA). Genotypes of HCV were also using the real-time polymerase chain reaction (RealTi*m*e HCV Genotype II; Abbott Molecular Inc., IL, USA). Fatty liver in sonography was defined as the presence of liver-renal echo contrast and bright liver [18] whereas splenomegaly was defined as the superior to the inferior axis of spleen more than 13 cm [19]. Liver stiffness was measured by TE, as reported elsewhere [20]. Briefly, each patient lied in the supine position with his or her right hand raised and truck bent to the left to maximize the right intercostal space. TE was performed by three physicians (HCC, PNC, and YCC) who each had experience of at least 500 measurements. The vibration wave of an M probe was generated and the liver stiffness and controlled attenuation parameter (CAP) were obtained in kPa and dB/m, respectively. Valid liver stiffness was identified by at least ten consecutive measurements with an interquartile range/median of kPa of less than 30% with an interquartile range of CAP of less than 40dB/m. The FIB-4 score of each participant was calculated using the equation, FIB-4 = [Age (years) x AST (U/L)]/ [platelet (10^9^/L) x √ALT (U/L)]. Stages of fibrosis were defined as follows; a FIB-4 score >3.25 or TE value ≥9.5 kPa for advanced fibrosis and a FIB-4 score ≤3.25 or a TE value <9.5 kPa for non-advanced fibrosis. Furthermore, lowest cutoff values of non-advanced fibrosis were set as a FIB-4 score of 1.45 or a TE value of 7.1kPa [14,15].

### Statistical analysis

Data were expressed as mean plus standard deviation or percentage. The correlation between FIB-4 scores and TE values was evaluated. One way ANOVA or the Kruskal–Wallis test was used to compare continuous variables among stages of fibrosis based on FIB-4 score alone, TE alone, and combined FIB-4 score/TE, to compare FIB-4 and TE by different age groups, and to compare the characteristics of subjects among FIB-4 or TE groups where appropriate. Multivariate logistic regression analysis was performed to search factors that were associated with advanced fibrosis defined by TE or FIB-4. Intraclass correlation coefficient was used to evaluate the reliability of FIB-4 and TE in evaluating fibrosis stages. All tests were two-tailed. A p value of less than 0.05 was considered to indicate statistically significance. Finally, data handling and analysis were carried out using SPSS software for Windows, version 17.0 (SPSS Inc., Chicago, IL).

## Results

### 1. Patients

A total of 905 who were positive for anti-HCV antibody voluntarily participated in surveillance activities. Following the exclusion of 401 subjects with undetectable HCV RNA and two subjects without valid liver stiffness measurement could be obtained, 502 subjects with detectable HCV RNA were enrolled in this investigation. Table 1 presents the baseline characteristics of the enrolled patients. They were predominantly female (62.2%) and genotype 2 was prevalent (50.6%).

**Table 1:** Baseline characteristics of 502 HCV viremic subjects.

Variable	
Age (years)	64.2 ± 9.9
Female/Male	312/190
Body mass index (kg/m^2^)	24.8 ± 4.1
HCV RNA (log_10_ IU/mL)	5.5 ± 1.3
HCV genotype	
1a	41
1b	105
2	254
3	2
6	77
Mixed	8
Undetermined	15
AST (U/L)	43.8 ± 32.6
ALT (U/L)	46.0 ± 41.3
Hemoglobin (g/dL)	13.7 ± 1.5
Platelet count (x10^9^/L)	198.3 ± 61.7
FIB-4 score	2.5 ± 1.9
Live stiffness (kPa)	7.9 ± 6.2

Mixed genotype: genotype 1+2: 1; 1a+1b: 1; 1b+2: 5; 4+6: 1.

### 2. Liver fibrosis staging

**Fig 1:**
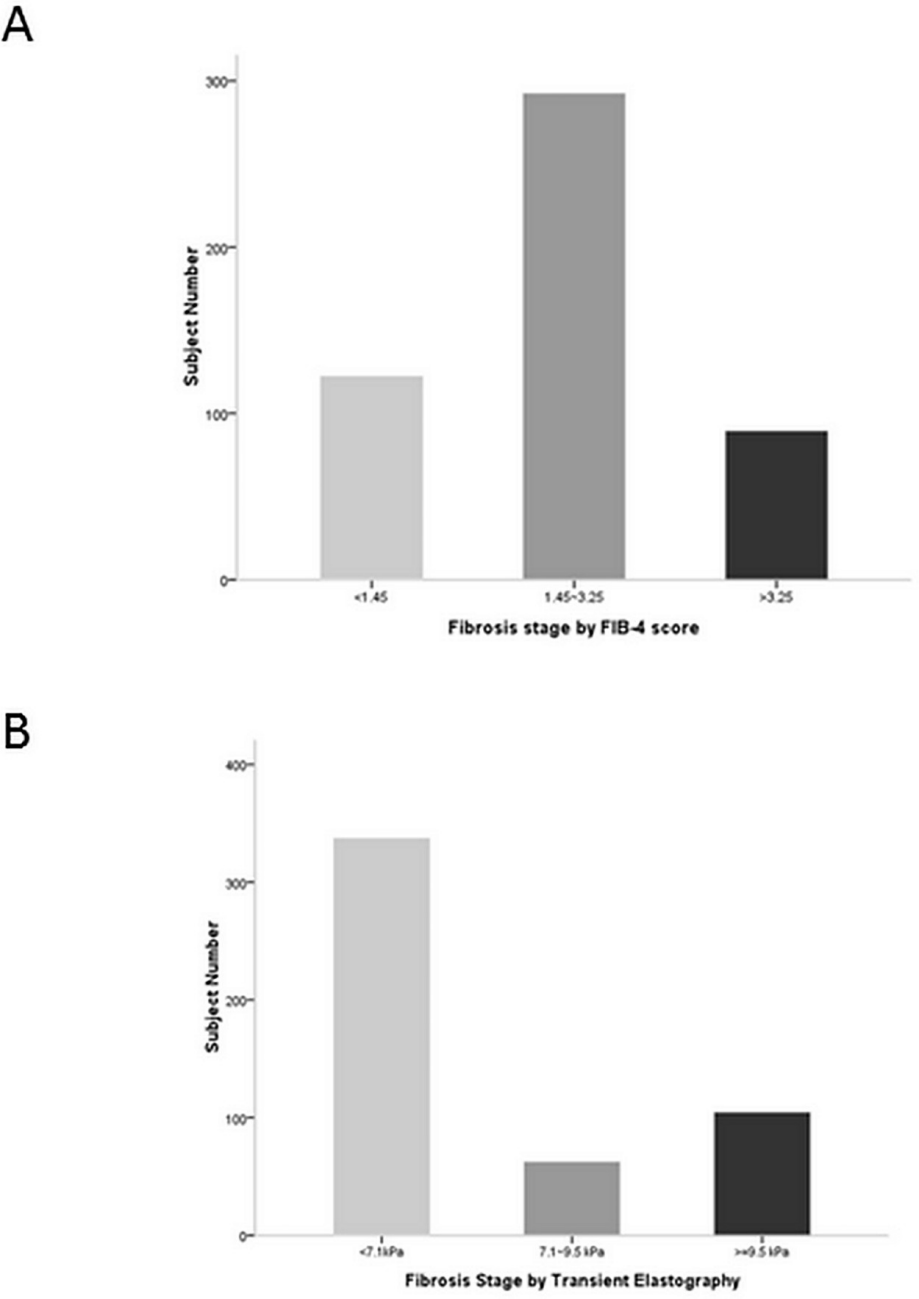
Fibrosis distribution. Fibrosis distribution of fibrosis severity according to (A) FIB-4 score or (B) Transient elastogrpahy.

The distributions of liver fibrosis among the same enrolled subjects that were determined from FIB-4 score and TE differed markedly (Fig 1). FIB-4 scores were <1.45 for 124 subjects (24.7%), between 1.45 and 3.25 for 290 subjects (57.8%), and ≥3.25 for 88 subjects (17.5%). TE scores were <7.1kPa for 337 subjects (67.1%), 7.1~9.5 kPa for 63 subjects (12.6%), and >9.5 kPa for 102 subjects (20.3%).

**Fig 2:**
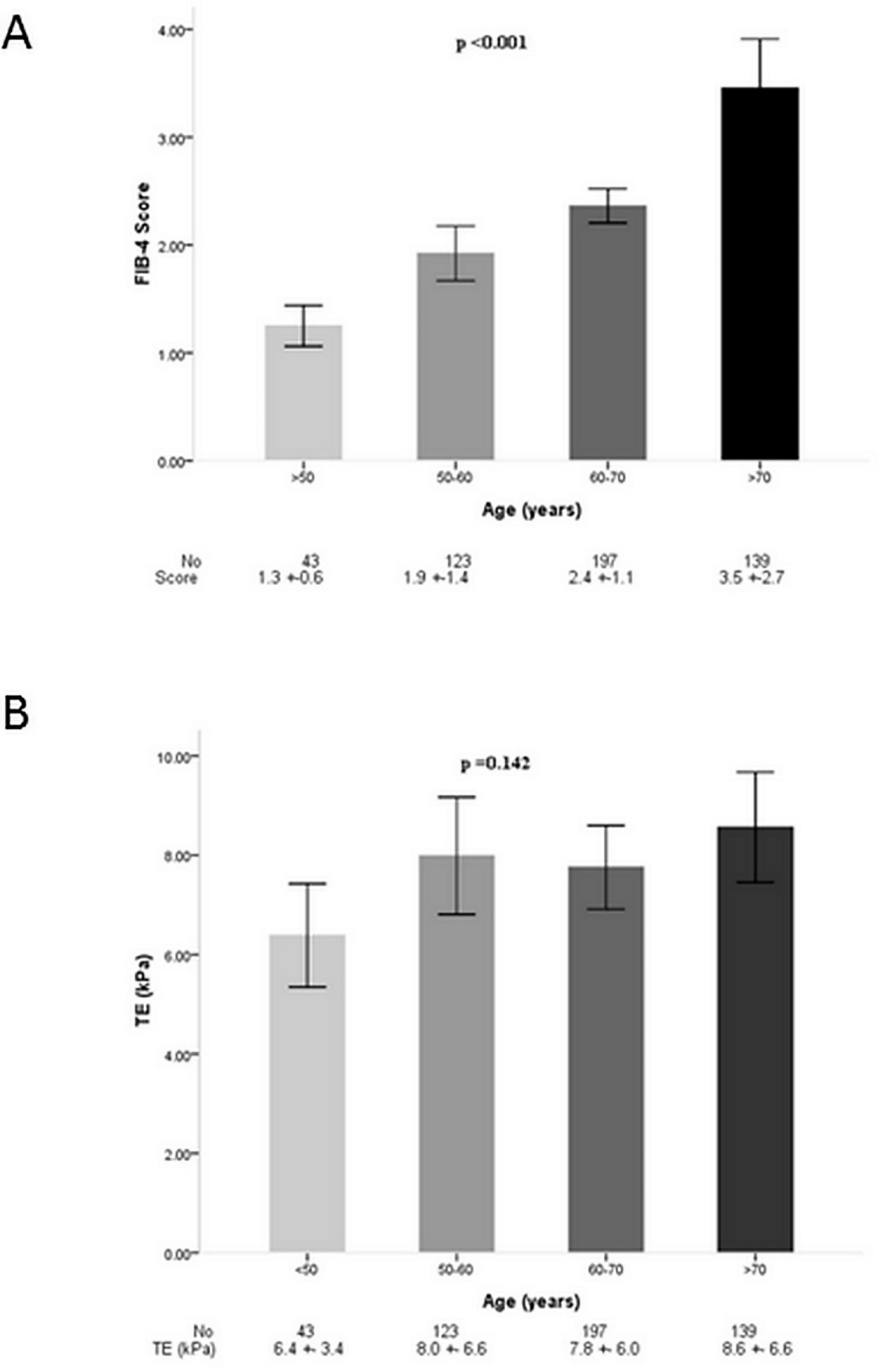
Fibrosis distribution of FIB-4 and TE according to age groups. Bar indicates mean values and lines indicated 95% of confidence interval. (A) FIB-4 distribution by age groups; (B) TE distribution by age groups.

Subjects fell into four age groups, which were <50 years, 50~60 years, 60~70 years, and >70 years. Moderate correlations between FIB-4 and TE among all subjects and among subjects in each age group were observed (Table 2). The strongest correlation was observed in subjects with ages 50~60 years (r = 0.655, p <0.001). Intraclass correlation efficient between FIB-4 and TE was 0.255 (p <0.001) that indicated the reliability of two methods was fair. Fig 2 demonstrates that the FIB-4 score increased proportionally with ages (p <0.001), but liver stiffness, measured by TE, did not (p = 0.142). Spots distribution of FIB-4 and TE according age groups were also different (S1 and S2 Figs).

**Table 2:** Correlation of FIB-4 and TE by age.

	All	<50 years	50~60 years	60~70 years	>70 years
**Subject No.**	502	43	123	197	139
**Coefficient**	0.458	0.412	0.655	0.346	0.502
**p values**	<0.001	0.006	<0.001	<0.001	<0.001

### 3. Comparisons of FIB-4 score with liver stiffness as determined by TE

A total of 152 subjects were classified into advanced fibrosis, as determined by either FIB-4 or TE, and were divided into three independent groups based on scoring methods of advanced fibrosis. They were FIB-4 (46 subjects), combined FIB-4/TE (43 subjects), and TE groups (63 subjects). The concordance rate of the two methods was 43/152 (28.3%) for advanced fibrosis. Sixty-three subjects (41.4%) would not have been identified as such if the FIB-4 score had been used alone. Similarly, the advanced fibrosis of 46 subjects (30.3%) would have been missed by using only TE. The univariate analysis revealed that subjects with advanced fibrosis in the TE group were younger, had higher platelet counts, higher body mass index (BMI), higher incidence of fatty liver, higher CAP values, higher incidence of splenomegaly, and lower FIB-4 scores (Table 3). Combined FIB-4/TE identified subjects with more severe liver diseases that were associated with higher liver stiffness and higher FIB-4 scores. Multivariate logistic regression analysis was performed to identify factors that were associated with the difference of FIB-4 and TE in advanced fibrosis subjects. Among the significant factors in univariate analysis, higher level of AST (odds ratio: 1.055, 95% CI: 1.008~1.015, p = 0.022), presence of fatty liver (odds ratio: 3.711, 95% CI: 1.079~12.758, p = 0.037), and presence of splenomegaly (odds ratio: 14.205, 95% CI: 2.598~77.678, p = 0.002) were the three independent factors associated with TE defined advanced fibrosis rather than FIB-4 defined advanced fibrosis.

**Table 3:** Comparisons of characteristics of fibrosis stages by FIB-4 or transient elastogrpahy.

Fibrosis stages*	FIB-4	FIB-4/TE	TE	
Advanced fibrosis				p value
Subject number	46	43	63	
Gender (female/male)	25/24	24/19	39/24	0.694
Age (years)	73.3 ± 7.2	70.4 ± 9.7	63.5 ± 8.9	<0.001
Body mass index (kg/m2)	24.1 ± 3.8	24.3 ± 3.5	26.3 ± 4.0	0.003
Waist circumference (cm)	81.2 ± 12.5	85.0 ± 17.0	86.2 ± 12.2	0.175
AST (U/L)	57.7 ± 33.6	93.9 ± 72.4	43.9 ± 17.3	<0.001
ALT (U/L)	59.0 ± 53.1	87.1 ± 86.8	50.2 ± 31.1	0.008
Platelet (103u/L)	135.7 ± 29.6	134.8 ± 43.1	205.3 ± 72.4	<0.001
Liver stiffness (kPa)	6.4 ± 1.6	19.1 ± 9.8	14.6 ± 8.0	<0.001
CAP (dB/m)	229.7 ± 40.4	230.1 ± 37.1	261.1 ± 52.2	<0.001
FIB-4 score	4.3 ± 0.8	6.0 ± 4.2	2.1 ± 0.7	<0.001
Fatty liver (yes/no)	8/38	13/30	36/27	<0.001
Splenomegaly (yes/no)	2/44	15/21	11/52	0.011
Genotype				0.116
1a	4	1	3	
1b	10	11	21	
2	24	25	28	
3	0	2	0	
6	8	4	6	
1b+2	0	0	2	
**Non-advanced fibrosis**				
Subject number	62	352	46	
Gender (female/male)	38/24	225/127	25/21	0.439
Age (years)	62.9 ± 9.2	62.5 ± 9.5	73.3 ± 7.2	<0.001
Body mass index (kg/m2)	26.3 ± 4.0	24.5 ± 4.1	24.1 ± 3.8	0.005
Waist circumference (cm)	86.1 ± 12.2	83.0 ± 41.1	81.3 ± 12.5	0.768
AST (U/L)	46.6 ± 27.5	35.8 ± 17.3	57.7 ± 33.6	<0.001
ALT (U/L)	51.9 ± 33.4	38.5 ± 26.8	59.0 ± 53.1	<0.001
Platelet (103u/L)	205.6 ± 72.7	213.1 ± 54.3	135.7 ± 29.6	<0.001
Liver stiffness (kPa)	14.8 ± 8.0	5.6 ± 1.4	6.4 ± 1.6	<0.001
CAP (dB/m)	260.7 ± 52.6	237.2 ± 45.2	229.7 ± 40.4	<0.001
FIB-4 score	2.1 ± 0.7	1.9 ± 0.6	4.3 ± 0.8	<0.001
Fatty liver (yes/no)	36/26	153/199	8/38	<0.001
Splenomegaly (yes/no)	11/52	12/340	2/44	<0.001
Genotype				0.262
1a	3	33	4	
1b	20	64	10	
2	27	178	24	
3	1	0	0	
6	6	59	8	
1+2	2	4	0	
4+6	0	1	0	
Undetermined	3	12	0	

*Advanced fibrosis: FIB-4 >3.25 and/or TE ≥9.5 kPa; Non-advanced fibrosis: FIB-4 ≤3.25 and/or TE <9.5 kPa.

Abbreviations: AST, aspartate aminotransferase; ALT, alanine aminotransferase; TE, transient elastography; CAP, controlled attenuation parameter.

For non-advanced fibrosis, the concordance rate between FIB-4 and TE was 76.5% (352/460). Subjects with non-advanced fibrosis in the TE group exhibited older, lighter BMI, lower platelet counts, lower TE and CAP values, higher FIB-4 scores, and lower incidence of fatty liver.

Fibrosis severity was categorized into three stages by TE values (Table 4). In TE defined advanced fibrosis, only 40.2% of subjects had FIB-4 >3.25. Among three groups, age, body mass index, HCV viral loads, and incidence of fatty liver were similar. As TE defined fibrosis stage progressed, significantly higher AST and ALT, higher waist circumference, lower platelet counts, higher FIB-4 score, higher CAP values, and higher incidence of splenomegaly were observed. The higher incidence of fatty liver and higher CAP values mainly observed in TE defined advanced fibrosis subjects with FIB-4 <1.45 (S1 Table).

**Table 4:** Comparisons of characteristics of fibrosis stages by transient elastogrpahy.

	Fibrosis Stages			
Variables	TE <7.1 kPa	TE 7.1~9.5 kPa	TE ≥9.5 kPa	p values
Subject number	337	63	102	
Gender (female/male)	211/126	40/23	61/41	0.853
Age (years)	63.7 ± 9.8	64.5 ± 10.6	66.0 ± 9.8	0.116
Body mass index (kg/m^2^)	24.5 ± 4.1	24.5 ± 3.8	25.5 ± 3.9	0.101
Waist circumference (cm)	80.8 ± 10.8	81.6 ± 10.5	84.8 ± 11.2	0.004
AST (U/L)	36.5 ± 20.5	48.2 ± 21.1	64.8 ± 54.6	<0.001
ALT (U/L)	38.4 ± 29.6	54.7 ± 37.8	65.9 ± 63.6	<0.001
Platelet (10^3^u/L)	207.0 ± 57.5	188.5 ± 55.4	176.0 ± 72.1	<0.001
HCV RNA (log_10_ IU/mL)	5.5 ± 1.3	5.6 ± 1.2	5.4 ± 1.3	0.555
FIB-4 score	2.1 ± 1.0	2.6 ± 1.2	3.7 ± 3.4	<0.001
FIB-4: >3.25, n (%)	32 (9.5)	15 (23.8)	41 (40.2)	
FIB-4: 1.45~3.25, n (%)	205 (60.8)	35 (55.6)	50 (49.0)	
FIB-4: <1.45, n (%)	100 (29.7)	13 (20.6)	11 (10.8)	
Fatty liver (yes/no)	139/198	25/38	47/55	0.633
CAP (dB/m)	234.7 ± 44.1	245.1 ± 46.7	248.4 ± 49.7	0.015
Splenomegaly (yes/no)	12/325	2/60	11/50	<0.001

## Discussion

In this investigation, FIB-4 and TE were utilized to determine the stages of fibrosis in a same CHC population in community. FIB-4 and TE yielded different patterns of the fibrosis distributions in the same population. The concordance rate of the two methods was low in diagnosis of advanced fibrosis. The characteristics of the subjects that were identified by FIB-4 and TE differed greatly. These results indicate that these two methods are not interchangeable and should be used clinically with care.

FIB-4 and TE yielded different distributions of the severity of fibrosis in a single study population. The measurement of liver stiffness by TE is based on machine-induced vibration that is barely affected by age. However, ALT level may affect the performance of TE [21, 22]. In this investigation, enrolled subjects exhibited low ALT levels (46.0 ± 41.3 U/L) that minimize the ALT effects on TE. The other factor that would affect TE performance is the presence of steatosis or fatty liver [23,24]. In this investigation, among three TE defined fibrosis stages, higher CAP values as fibrosis stages became severe, especially in those subjects with FIB-4 <1.45 in TE defined advanced fibrosis. These results indicated the possible role of steatosis in the measurement of liver stiffness by TE, especially combined with low FIB-4 score subjects.

Liver biopsy is currently the gold standard clinical method for evaluating fibrosis stage. However, liver histological evaluations cannot be performed in the community. Non-invasive methods, such as FIB-4 and TE, are easier to perform than a liver biopsy and can be instantly interpreted. Vallet-Pichard et al. found that FIB-4 had positively predicted advanced fibrosis with 82.1% concordance with liver biopsy results [14]. Similarly, a study that compared TE with liver biopsy found that the former had a high rate of positive prediction of advanced fibrosis (19) [15]. However, the findings by FIB-4 and TE of advanced fibrosis were concordant for only 28.3% of CHC subjects in this study. Age severely influenced FIB-4 score, but not TE. The mean age (64.2 ± 9.9 years) of the subjects in this study exceeded that in the study of Vallet-Pichard et al. (44 ± 12 years). About 70% of enrolled subjects were older than 60 years in this investigation. The subjects who were diagnosed with advanced fibrosis by FIB-4 were older than those diagnosed with by TE (73.3 ± 7.2 years vs. 63.5 ± 8.9 years, p <0.001). Greater age is not necessarily associated with more severe fibrosis and is an important factor in the failure of FIB-4 calculations [14]. In this investigation, about 60% of the cases of advanced fibrosis that were identified using TE had FIB-4 score ≤ 3.25. Presence of splenomegaly did associate with TE-defined advanced fibrosis rather than FIB-4 defined advanced fibrosis. The correlation between FIB-4 and TE was greatest for participants of ages 50~60 years. All of these results suggest that age is an important determinant of FIB-4 score but not TE, and FIB-4 calculations should be cautiously used to evaluate the stage of fibrosis in subjects older than 60 years. In addition, TE has been proved to be effective for predicting the long-term outcomes of CHC [25]. Lines of evidence showed that some extrahepatic factors including obesity, impaired glucose metabolism, and steatosis accelerate disease progression of CHC [26–29]. Presence of fatty liver was strongly associated with TE-defined advanced fibrosis rather than FIB-4 defined advanced fibrosis. In those subjects with TE defined advanced fibrosis, higher CAP values and increased incidence of fatty liver was observed in subjects FIB-4 <1.45 indicated that TE may have the advantage over than FIB-4 to detect the involvement of non-alcoholic fatty liver disease in fibrosis progression of chronic hepatitis C. TE is probably better than FIB-4 for evaluating liver fibrosis in CHC subjects in community.

Potential limitations of this study should be addressed. First, all comparisons were performed based on defined cutoff values of the non-invasive methods, not based on liver histology. Second, the clinical significance of intermediate FIB-4 scores or TE values seems to be inconclusive according to current evidence [14,15]. Third, detail medical histories, covering diabetes mellitus, dyslipidemia, and lipid or sugar profiles, were not obtained. Any potential association between fatty liver and CAP in subjects with advanced or mild fibrosis could not be analyzed in this community-based study.

In summary, FIB-4 and TE defined different fibrosis stages in the same CHC population in community. Age deeply affected the FIB-4 evaluation. TE had the advantages over than FIB-4 in strong association with splenomegaly and in detecting the contributing role of non-alcoholic fatty liver disease in subjects with advanced fibrosis.

## Supporting information

S1 File Date set in SPSS format. (SAV)

S1 Table Characteristics of subjects with different ranges of FIB-4 scores in defined fibrosis stages by TE. (DOCX)

S1 Fig The scatter plot of FIB-4. (TIF)

S2 Fig The scatter plot of TE. (TIF)

## Abbreviations

HCV: hepatitis C virus
DAA: direct acting antiviral
CHC: chronic hepatitis C
TE: transient elastography
AST: aspartate aminotransferase
ALT: alanine aminotransferase
CAP: controlled attenuation parameter
