# Supplementary material for: Comparison of FIB-4 and transient elastography in evaluating liver fibrosis of chronic hepatitis C subjects in community
Source: PLoS One. 2018 Nov 7;13(11):e0206947. doi: 10.1371/journal.pone.0206947 (PMC6221348; doi:10.1371/journal.pone.0206947)
Supplement: S1 Table — (DOCX) [file pone.0206947.s002.docx]

**S1 Table. Characteristics of subjects with different ranges of FIB-4 scores in defined fibrosis stages by TE**

|  | **TE ≥9.5 kPa** | | |  | | **TE 7.1~9.5 kPa** | | | | | | |  | | | **TE <7.1 kPa** | | | | | |  | | | |  |  |  |
| --- | --- | --- | --- | --- | --- | --- | --- | --- | --- | --- | --- | --- | --- | --- | --- | --- | --- | --- | --- | --- | --- | --- | --- | --- | --- | --- | --- | --- |
|  | **FIB-4 Score** | | |  | | **FIB-4 Score** | | | | | |  | | | **FIB-4 Score** | | | | | | |  | | |  |  |  |  |
| **Variables** | <1.45 | 1.45~3.25 | ≥3.25 | *p* | | <1.45 | 1.45~3.25 | | ≥3.25 | | *p* | | | <1.45 | | | 0..45~3.25 | | ≥3.25 | | | *p* | | | | |  |  |
| Subject number | 11 | 50 | 41 |  | 13 | | | 35 | | 15 | | |  | 100 | | | | 205 | | 32 | | | |  | | | |  |
| Gender (female/male) | 8/3 | 30/20 | 23/18 | 0.607 | 7/6 | | | 21/14 | | 12/3 | | | 0.291 | 66/34 | | | | 131/74 | | 14/18 | | | | 0.064 | | | |  |
| Age (years) | 56.2 ± 10.3 | 64.0 ± 8.1 | 71.0 ± 8.7 | <0.001 | 55.9 ± 10.0 | | | 63.7 ± 8.5 | | 74.0 ± 8.3 | | | <0.001 | 56.7 ± 10.0 | | | | 65.6 ± 7.8 | | | 73.1 ± 6.7 | | <0.001 | | | | | |
| Body mass index (kg/m^2^) | 25.8 ± 3.3 | 26.5 ± 4.1 | 24.3 ± 3.6 | 0.027 | 25.8 ± 4.8 | | | 24.1 ± 3.1 | | 24.4 ± 4.3 | | | 0.603 | 25.0 ± 5.4 | | | | 24.5 ± 3.5 | | 24.0 ± 3.6 | | | | 0.448 | | | |  |
| Waist circumference (cm) | 81.0 ± 15.7 | 87.4 ± 11.3 | 82.8 ± 9.1 | 0.070 | 84.0 ± 13.7 | | | 81.6 ± 9.1 | | 79.6 ± 10.8 | | | 0.545 | 81.6 ± 10.8 | | | | 80.1 ± 10.4 | | 82.0 ± 13.1 | | | | 0.545 | | | |  |
| AST (U/L) | 31.5 ± 8.9 | 49.9 ± 29.4 | 91.8 ± 71.5 | <0.001 | 38.7 ± 12.8 | | | 46.4 ± 19.8 | | 60.7 ± 24.9 | | | 0.015 | 28.3 ± 11.9 | | | | 37.5 ± 17.9 | | 56.3 ± 36.8 | | | | <0.001 | | | |  |
| ALT (U/L) | 38.1 ± 17.8 | 55.2 ± 35.6 | 86.5 ± 88.3 | 0.015 | 52.3 ± 34.2 | | | 49.5 ± 29.5 | | 68.9 ± 54.1 | | | 0.733 | 31.6 ± 20.3 | | | | 39.2 ± 27.7 | | 54.3 ± 52.0 | | | | 0.033 | | | |  |
| Platelet (10^3^u/L) | 286.8 ± 122.7 | 188.0 ± 41.3 | 131.4 ± 40.9 | <0.001 | 258.2 ± 48.9 | | | 184.8 ± 38.0 | | 136.6 ± 24.5 | | | <0.001 | 260.9 ± 56.9 | | | | 191.6 ± 36.7 | | 136.8 ± 32.7 | | | | <0.001 | | | |  |
| HCV RNA (log_10_ IU/mL) | 5.4 ± 1.6 | 5.5 ± 1.5 | 5.2 ± 1.1 | 0.665 | 5.9 ± 1.0 | | | 5.5 ± 1.2 | | 5.5 ± 1.3 | | | 0.539 | 5.6 ± 1.2 | | | | 5.4 ± 1.4 | | 5.5 ± 1.0 | | | | 0.260 | | | |  |
| Fatty liver (yes/no) | 8/3 | 27/23 | 12/29 | 0.011 | 7/6 | | | 12/22 | | 5/10 | | | 0.488 | 49/51 | | | | 86/119 | | 4/28 | | | | 0.001 | | | |  |
| CAP (dB/m) | 287.4 ± 50.1 | 255.1 ± 52.3 | 229.8 ± 49.7 | 0.001 | 251.8 ± 47.7 | | | 244.5 ± 47.1 | | 240.7 ± 45.6 | | | 0.823 | 236.8 ± 44.7 | | | | 235.3 ± 45.0 | | 224.7 ± 44.1 | | | | 0.360 | | | | |
| Splenomegaly (yes/no) | 2/11 | 9/41 | 12/29 | 0.412 | 0/13 | | | 2/33 | | 2/15 | | | 0.438 | 4/96 | | | | 6/199 | | 2/30 | | | | 0.616 | | | | |
