# Supplementary figures and images for: Comparison of FIB-4 and transient elastography in evaluating liver fibrosis of chronic hepatitis C subjects in community
Source: PLoS One. 2018 Nov 7;13(11):e0206947. doi: 10.1371/journal.pone.0206947 (PMC6221348; doi:10.1371/journal.pone.0206947)

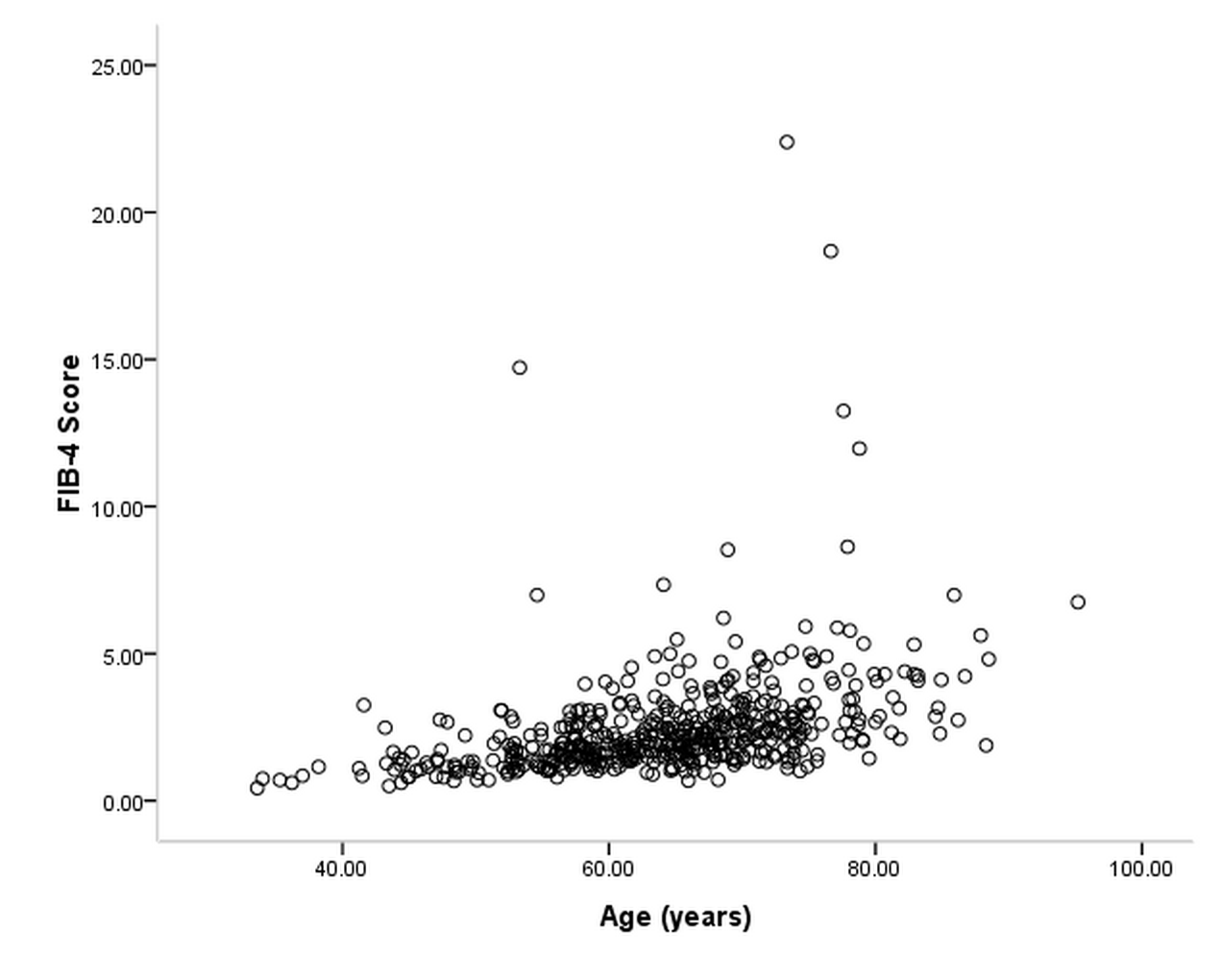

Supplement: S1 Fig — (TIF) [file pone.0206947.s003.tif]

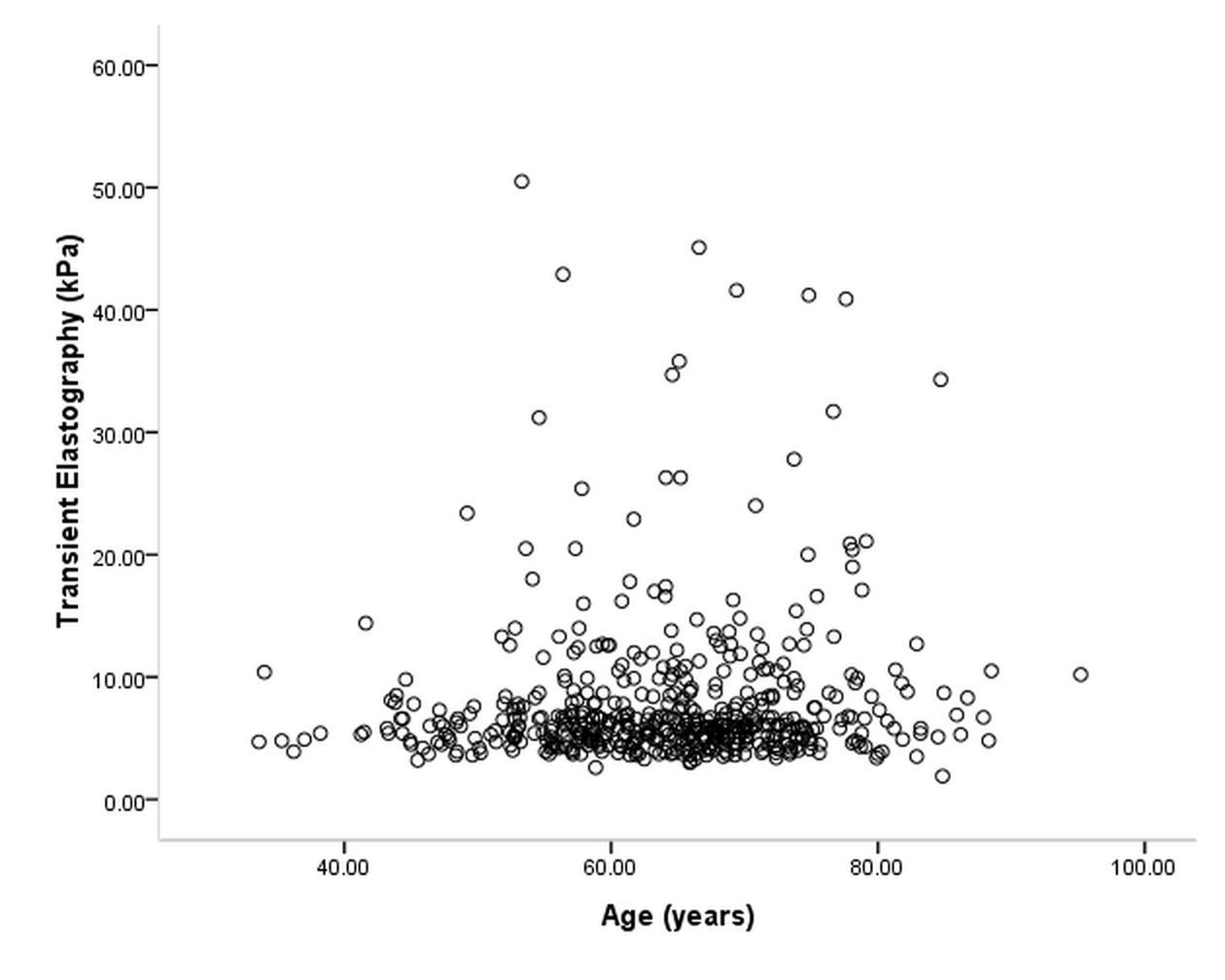

Supplement: S2 Fig — (TIF) [file pone.0206947.s004.tif]
